# Calendar

**DOI:** 10.1155/S1463924682000236

**Published:** 1982


					Journal of Automatic Chemistry, Volume 4, Number 2 (April-June 1982), page 81
Calendar
Editor's Note:
Organizers of conferences, seminars, etc.
should send details for inclusion in the
calendar as soon as the relevant infor-
mation is available and not later than three
months before the event.
April 1982
Analytica 82
International Conference on Biochemical
and Instrumental Analysis, and Trade
Fair. To be held at the Munich Trade Fair
Grounds from 27 to 30 April 1982.
Contact Miinchener Messe-and Ausstel-
lungsgesellschaft mbH, Messegelnde,
Postfach 12 10 09, D 8000 Miinchen 12,
FR Germany.
14th Annual Symposium on Advanced
Analytical Concepts for the Clinical
Laboratory
Sponsored by the American Association
for Clinical Chemistry and Oak Ridge
National Laboratory. To be held on 29
and 30 April at Riverside Motor Lodge,
Gatlinburg, Tennessee.
Contact Carl A. Burtis, Oak Ridge
National Laboratory, PO Box X, Oak
Ridge, Tennessee 37830, USA.
May 1982
Automated Combination Techniques with
Mass Spectrometry
Organized by the Automatic Methods
Group and Western Region of the Royal
Society of Chemistry. To be held on 12
May at Messrs W. D. and H. O. Wills,
Bristol, UK.
Contact C. J. Jackson, HSE Labs.,
403 Edgware Road, London NW2 6LN.
June 1982
Achema 82: The International Meeting of
Chemical Engineering
Organized by Dechema, Achema 82
consists of the following conferences:
20th Chemical Engineering Exhibition
and Congress, Formal Meeting of the
Gesellschaft Deutscher Chemiker,
Formal Meeting of the VDI Society for
Process Engineering and Chemical
Engineering and the International Social
Security Association International
Colloquium. To be held from 6-12 June
at the Fairgrounds and Halls of the
Frankfurte Messe GMBH, FR Germany.
Contact Dechema, PO Box 970146, D 6000
Frankfurt am Main, FR Germany.
'Chemistry Days in Analytical Chemistry'
(Kemistdagar Analytisk Kemi)
Organized by the Analytical Section of
the Swedish Chemical Society. To be held
from 15-18 June in Lund, Sweden.
Contact the Swedish Chemical Society,
Upplandsgatan 6A, 1 tr, S-111 23
Stockholm.
'Modern Methods of Food Analysis': a
Basic Science Symposium of the Institute
of Food Technologists
To be held from 17-18 June in New
Orleans, USA.
Contact Dr Kent K. Stewart, Department
of Food Science & Technology, Virginia
Polytechnic and State University,
Blacksburg, Virginia 24061, USA.
Flow Analysis II
Organized by the Analytical Section of
the Swedish Chemical Society. To be held
from 18 to 21 June at the Hotel Sparta,
Lund, Sweden.
Contact 'Flow Analysis II', c/o The
Swedish Chemical Society, Upplandsgatan
6A, tr, S-111 23 Stockholm.
International Conference on Chromato-
graphy and Mass Spectrometry in the
Biomedical Sciences
Organized by the Italian Group for Mass
Spectrometry and the International
Scientific Center. To be held from 21 to 23
June at Bordighera, Italy.
Contact Dr Alberto Frigerio, c/o Istituto
di Richerche Farmacologiche 'Mario
Negri', Via Eritrea 62, I 20157 Milan,
Italy.
July 1982
Swansea Summer School of Automatic
Chemical Analysis
To be held from 11-16 July at University
College, Swansea, UK.
Contact Professor D. Betteridge, Chemistry
Department, University College, Singleton
Park, Swansea SA2 8PP, UK.
September 1982
14th Japanese Congress on Clinical
Laboratory Automation
Organized by the Japanese Society of
Clinical Laboratory Automation. To be
held from 10 to 11 September in Kobe,
Japan.
Contact Dr Koki Motege, c/o Tokyo
Clinical Laboratory, 9-3 Nakamarucho,
Habashi-ku, Tokyo.
Microcomputers in Chemical
Instrumentation
Intensive five-day course organized by
the Chemistry Microprocessor Unit of
the Chemistry Department at Imperial
College, London. To be held from 20 to
24 September at Imperial College.
Contact Dr Nick Goddard, Chemistry
Microprocessor Unit, Department of
Chemistry, Imperial College, London
SW7 2A Y.
October 1982
29th Japanese Congress for Clinical
Pathologists
Organized by the Japanese Society of
Clinical Pathologists. To be held from
22 to 24 October in Gifu, Japan.
Contact Dr Nozomu Kosakai, c/o
Juntendo University Medical School,
Department of Clinical Pathology, 2-1-1
Hongo, Bunkyo-ku, Tokyo 113.
81

				

